# Partnering With Patients, Caregivers, and Clinicians to Determine Research Priorities for Concussion

**DOI:** 10.1001/jamanetworkopen.2023.16383

**Published:** 2023-06-07

**Authors:** Martin H. Osmond, Elizabeth Legace, Peter J. Gill, Rhonda Correll, Katherine Cowan, Jennifer E. Dawson, Randene Duncan, Erin Fox, Kanika Gupta, Ash T. Kolstad, Lisa Marie Langevin, Colin Macarthur, Rosemary Macklem, Kinga Olszewska, Nick Reed, Roger Zemek

**Affiliations:** 1 Department of Pediatrics, Faculty of Medicine, University of Ottawa, Ottawa, Ontario, Canada; 2 Children’s Hospital of Eastern Ontario Research Institute, Ottawa, Canada; 3 Department of Pediatrics, The Hospital for Sick Children, University of Toronto, Toronto, Ontario, Canada; 4 Child Health Evaluative Sciences, SickKids Research Institute, Toronto, Ontario, Canada; 5 James Lind Alliance, Southampton, United Kingdom; 6 Patient Partner, Children’s Hospital of Eastern Ontario, Ottawa, Canada; 7 Sport Injury Prevention Research Centre, Faculty of Kinesiology, University of Calgary, Calgary, Alberta, Canada; 8 Alberta Children’s Hospital Research Institute for Child and Maternal Health, Alberta Children’s Hospital, Calgary, Canada; 9 currently a graduate student at the University of Calgary, Calgary, Alberta, Canada; 10 Department of Psychology, Cumming School of Medicine, University of Calgary, Calgary, Alberta, Canada; 11 Faculty of Arts, University of Calgary, Calgary, Alberta, Canada; 12 Department of Occupational Science and Occupational Therapy, University of Toronto, Toronto, Ontario, Canada

## Abstract

**Importance:**

Identifying research priorities of patients with concussion, their caregivers, and their clinicians is important to ensure future concussion research reflects the needs of those who will benefit from the research.

**Objective:**

To prioritize concussion research questions from the perspectives of patients, caregivers, and clinicians.

**Design, Setting, and Participants:**

This cross-sectional survey study used the standardized James Lind Alliance priority-setting partnership methods (2 online cross-sectional surveys and 1 virtual consensus workshop using modified Delphi and nominal group techniques). Data were collected between October 1, 2020, and May 26, 2022, from people with lived concussion experience (patients and caregivers) and clinicians who treat concussion throughout Canada.

**Exposures:**

The first survey collected unanswered questions about concussion that were compiled into summary questions and checked against research evidence to ensure they were unanswered. A second priority-setting survey generated a short list of questions, and 24 participants attended a final priority-setting workshop to decide on the top 10 research questions.

**Main Outcomes and Measures:**

Top 10 concussion research questions.

**Results:**

The first survey had 249 respondents (159 [64%] who identified as female; mean [SD] age, 45.1 [16.3] years), including 145 with lived experience and 104 clinicians. A total of 1761 concussion research questions and comments were collected and 1515 (86%) were considered in scope. These were combined into 88 summary questions, of which 5 were considered answered following evidence review, 14 were further combined to form new summary questions, and 10 were removed for being submitted by only 1 or 2 respondents. The 59 unanswered questions were circulated in a second survey, which had 989 respondents (764 [77%] who identified as female; mean [SD] age, 43.0 [4.2] years), including 654 people who identified as having lived experience and 327 who identified as clinicians (excluding 8 who did not record type of participant). This resulted in 17 questions short-listed for the final workshop. The top 10 concussion research questions were decided by consensus at the workshop. The main research question themes focused on early and accurate concussion diagnosis, effective symptom management, and prediction of poor outcomes.

**Conclusions and Relevance:**

This priority-setting partnership identified the top 10 patient-oriented research questions in concussion. These questions can be used to provide direction to the concussion research community and help prioritize funding for research that matters most to patients living with concussion and those who care for them.

## Key Points

**Question:**

What are the most important research priorities in concussion from the perspective of patients, caregivers, and clinicians?

**Findings:**

This survey study used 2 cross-sectional surveys and a consensus workshop with a nominal group technique to gather perspectives from patients, caregivers, and clinicians to identify the top 10 concussion research questions. The main question themes focused on accurate concussion diagnosis, effective symptom management, and prediction of poor outcomes.

**Meaning:**

This research priority list provides a guide to the concussion research community and can help prioritize funding for research that matters most to those living with concussion and those who care for them.

## Teaser

This cross-sectional survey study uses priority-setting partnership methods to identify the most important unanswered research questions in concussion from the perspectives of patients, caregivers, and clinicians.

## Introduction

Concussion is a mild traumatic brain injury affecting 2% of the population each year.^1,2,3^ Up to 30% of patients experience prolonged symptoms lasting more than 1 month, including headache, visual problems, balance deficits, sleep disruptions, fatigue, and difficulties with cognition.^4,5,6^ As a result, concussion often leads to school and work absences, less physical activity, mental health sequelae, and altered quality of life.^6,7,8,9,10,11,12,13^ Several national^14,15,16^ and international^17^ clinical guidelines, however, have noted that many aspects of concussion diagnosis and management lack research evidence to guide recommendations. To address this, multidisciplinary research networks, including the Canadian Concussion Network, are being established to generate high-quality research.

In this context, an important first step is to establish research priorities. In the past, research priorities were typically set by academic researchers, pharmaceutical companies, medical device industries, or funding bodies. More recently, the Strategy for Patient-Oriented Research in Canada^18^ and the Patient-Centered Outcomes Research Institute in the US^19^ have encouraged researchers to seek out the perspectives of those with lived experience to determine important and unanswered questions. This approach leads to priority setting that is relevant to patients and more readily implemented in clinical practice.^18,19^

The James Lind Alliance (JLA) brings together patients, caregivers, and clinicians to form priority-setting partnerships (PSPs) to identify and prioritize important unanswered questions in health care.^20^ The JLA process is grounded in principles such as equal involvement of patients, caregivers, and clinicians; transparency; mandatory sharing of an audit trail; and exclusion of researchers and groups with competing interests from the prioritization process.^20^ The JLA-PSP method has engaged patients in setting research priorities for more than 150 discrete health conditions.^21^ Our aim was to conduct a research prioritization study using JLA-PSP methods to identify the most important unanswered questions in concussion from the perspectives of patients, caregivers, and clinicians.

## Methods

### Study Design

In this cross-sectional survey study, we used standardized JLA-PSP methods, which draw on modified Delphi and nominal group techniques.^22^ Steps included establishing a steering group for the partnership; identifying research uncertainties through a national cross-sectional survey; summarizing top-ranked questions and determining their inclusion via evidence verification; interim priority setting through a second national cross-sectional survey; and a priority-setting workshop to rank the questions in order of priority. The study followed the Reporting Guideline for Priority Setting of Health Research (REPRISE) and the American Association of Public Opinion Research (AAPOR) Standards Best Practices. The project was conducted from October 1, 2020, to May 26, 2022. Research ethics board approval was obtained from the Children’s Hospital of Eastern Ontario. Potential respondents were informed of the risks and benefits of the study in the survey introduction and informed that by completing the survey they were agreeing to participate in the study.

### Scope

This pan-Canadian PSP sought individuals diagnosed with or suspected of having a concussion or mild traumatic brain injury, as well as caregivers of people with concussion and clinicians of all disciplines who treat concussion. We used the 5th Consensus Statement on Concussion in Sport to define concussion as “a traumatic brain injury induced by biomechanical forces” that results in 1 or more symptoms in the clinical domains of somatic, cognitive, emotional and/or behavioral, or sleep.^17^ Patients of all ages, any mechanism of injury, any symptom burden, and any duration of postinjury symptoms were included. The scope of questions included those relating to diagnosis, treatment, management, prognosis, and prevention of concussion. We excluded questions related to moderate or severe brain injury, brain injury not caused by physical trauma, and health care resourcing.

### Steering Group

The steering group, established to oversee the operations of the PSP, included 6 patients with concussion, 1 caregiver, 6 clinicians who treat concussion, and 1 concussion stakeholder organization member.^23^ The PSP lead (M.H.O.) and senior JLA advisor (K.C.) were coleaders of the steering group. The steering group met monthly and was responsible for (1) finalizing the scope; (2) designing and piloting the surveys; (3) overseeing a plan to publicize the online surveys; (4) assisting with classification and verification of summary questions; and (5) identifying the short list of questions for the final workshop.

### Step 1: Gathering Unanswered Questions (Survey 1)

To gather unanswered research questions related to concussion, the steering group developed a bilingual (English and French), anonymous, web-based survey. Study data were encrypted and securely collected on REDCap^24^ and stored on a password-protected laptop in a locked room. The survey (eFigure 1 in Supplement 1) was composed of 5 open-ended questions designed to elicit free-text questions from patients, caregivers, and clinicians concerning diagnosis, treatment, management, prognosis, and prevention of concussion. The survey collected participant demographics (age, sex, race and ethnicity or cultural background, language, place of residence), patient characteristics (number of concussions, length of symptoms, mechanism), and role of the health care professionals or clinicians (eg, physicians, nurses, physiotherapists, occupational therapists, neuropsychologists, athletic therapists). Respondents’ demographic characteristics were tracked to subsequently target underrepresented groups. The steering group advised on the survey wording, layout, images, and font size and/or type to improve accessibility for those with concussion. The survey was pilot tested on 14 people for comprehension, clarity, and time for completion. A paper version of the survey or telephone completion was also offered.

The survey was open from February 16 to May 17, 2021, to patients of all ages with concussion, caregivers, and clinicians across Canada. The survey was distributed broadly through websites, emails, newsletters, and social media by strategic partners (professional health care organizations, sports organizations, patient-oriented concussion groups, and brain injury stakeholders). Participant characteristics were analyzed using descriptive statistics.

### Step 2: Generating Summary Research Questions and Evidence Verification

Questions from survey 1 were categorized into themes by the steering group through an iterative process. Out-of-scope questions were removed. Patient-clinician dyads formulated summary questions. The steering group reviewed summary questions, and the final wording of the questions was settled by consensus.

Evidence checking was completed by a team convened by one of us (L.M.L.) who provided perspectives from specialized fields, including knowledge translation, research, public policy, and clinical practice. These information specialists examined recent research, databases, and current guidelines. For pragmatic reasons, focused searches to identify systematic reviews, primary research, clinical guidelines, and consensus statements were conducted. We searched the MEDLINE, Embase, CINAHL, Epistemonikos, and Cochrane Central Register of Controlled Trials databases from 2010 onward for English language publications. Questions were classified as unanswered if the search found no systematic review, if recent systematic reviews found the question unanswered or yielded contradictory results, if there was a lack of primary research, or if there was insufficient evidence in the 5th International Consensus Statement on Concussion in Sport^17^ or national concussion evidence-based practice guidelines.^14,15,16^ Unanswered questions were included for the interim priority-setting survey. Questions considered answered by previous research were compiled separately.

### Step 3: Interim Priority Setting (Survey 2)

A second anonymous online survey (available in English and French) shared the long list of unanswered summary questions (finalized in step 2) and requested that participants choose the 10 most important questions from their perspective. The order in which the list of questions was presented was randomized weekly to minimize bias. The survey was pilot tested on 5 people for comprehension, clarity, and time for completion. Demographic data were also collected in survey 2, which was open from January 10 to April 10, 2022; promotion, distribution, and data storage paralleled that of survey 1. A scoring system was used to analyze the data. One point was assigned for each question in a respondent’s top 10 list. Points were tallied separately for 2 distinct groups: (1) those with lived experience (eg, patients, caregivers) and (2) clinicians who treat concussion. To ensure equal influence for each group, questions were ranked from highest to lowest for each group and the 2 scores were summed to give a total score which determined the combined rank order for each question. The steering group reviewed the final rankings and reached consensus on the short list of questions for the priority-setting workshop.

### Step 4: Final Priority-Setting Workshop

A workshop consisting of 2 half-day sessions (May 25 and 26, 2022) and conducted through video teleconference (Zoom Video Communications) was used to rank the final list of unanswered research questions by order of importance and generate a final top 10 list. Twenty-four participants were invited to attend (from surveys 1 and 2 and personal networks). The workshop sought equal representation from those with lived experience and clinicians and sought diversity in age, sex, geography, concussion mechanism, and clinician expertise. Two weeks prior to the workshop, participants were sent the short list of 17 final questions via email and post. The workshop was led by a senior JLA advisor (K.C.) and used a modified nominal group technique.^25,26^ Three additional JLA advisors facilitated breakout sessions to ensure balanced participation and respectful discussion across participants. To enable a wide exchange of knowledge and consider all perspectives, participants rotated through different combinations of breakout groups. Consensus on the top 10 questions was reached using established JLA methods.^22^ Degree of participant engagement was assessed through a postworkshop survey.

## Results

### Gathering Unanswered Research Questions (Survey 1)

Two hundred forty-nine participants completed survey 1 (Table 1). Of these, 145 respondents (58%) had lived experience of concussion and 104 (42%) were clinicians. Mean (SD) age was 45.1 (16.3) years. One hundred fifty-nine respondents (64%) identified as female and 85 (34%) as male, with 5 (2%) preferring not to answer. Self-reported race and ethnicity or cultural background included Asian (14 [6%]), Black (1 [0.4%]), Indigenous (2 [1%]; ie, First Nations, Métis, or Inuk or Inuit), Latino (1 [0.4%]), Middle Eastern (5 [2%]), and White (218 [88%]). Respondents were from across Canada except the 3 northern territories. Of the 138 respondents who were patients with concussion, 59 (43%) had had 3 or more concussions, 69 (50%) experienced concussion symptoms lasting longer than 1 year, and 77 (56%) reported ongoing concussion symptoms. Of all 195 injury mechanisms reported, sports injuries were the most common (83 [43%]).

**Table 1.** Characteristics of Participants^a^

Characteristic	Survey 1 (n = 249)	Survey 2 (n = 989)
Type of participant^b^		
Patient	138 (55)	511 (52)
Caregiver	17 (7)	8 (1)
Family member or friend	99 (40)	99 (10)
Teacher or coach	50 (20)	24 (2)
Other	19 (8)	12 (1)
Health care professional	104 (42)	327 (33)
Physician	46 (18)	94 (10)
Physiotherapist	10 (4)	89 (9)
Athletic therapist	10 (4)	32 (3)
Occupational therapist	6 (2)	21 (2)
Neuropsychologist	5 (2)	11 (1)
Nurse	4 (2)	11 (1)
Chiropractor	3 (1)	3 (0.3)
Social worker	2 (1)	2 (2)
Other	18 (7)	62 (6)
Did not answer	NA	8
Age, mean (SD), y	45.1 (16.3)	43.0 (4.2)
Sex		
Female	159 (64)	764 (77)
Male	85 (34)	206 (21)
Other	0	1 (0.1)
Prefer not to say	5 (2)	18 (2)
Race or ethnic or cultural background		
Asian (East, Southeast, or South Asian)	14 (6)	53 (5)
Black (African or Afro-Caribbean)	1 (0.4)	11 (1)
Indigenous (First Nations, Métis, or Inuk/Inuit)	2 (1)	21 (2)
Latino (Latin American or Hispanic descent)	1 (0.4)	12 (1)
Middle Eastern	5 (2)	11 (1)
White (North American or European descent)	218 (88)	882 (89)
Other^c^	3 (1)	7 (1)
Prefer not to say	5 (2)	21 (2)
Primary language spoken at home		
English	201 (81)	817 (83)
French	39 (16)	23 (2)
Other	3 (1)	138 (14)
Prefer not to say	6 (2)	11 (1)
Province or territory of residence		
British Columbia	19 (8)	79 (8)
Prairies (Alberta, Manitoba, or Saskatchewan)	67 (27)	202 (20)
Ontario	99 (40)	433 (44)
Quebec	43 (17)	144 (15)
Maritimes (Nova Scotia, New Brunswick, or Prince Edward Island)	7 (3)	56 (6)
Newfoundland and Labrador	4 (2)	5 (1)
Territories (Northwest Territories, Nunavut, or Yukon)	0	0
Not answered	10 (4)	70 (7)
What best describes where you live?		
Urban	187 (75)	790 (80)
Rural	57 (23)	185 (19)
Not answered	5 (2)	14 (1)
How many concussions have you had?^d^		
1	35 (25)	155 (30)
2	27 (20)	100 (20)
≥3	59 (43)	226 (44)
Do not know or unsure	17 (40)	30 (6)
At what age did you have your concussion(s)?^d^		
0-5 y	13 (9)	25 (5)
6-10 y	20 (14)	59 (12)
11-15 y	30 (22)	130 (25)
16-20 y	47 (34)	152 (30)
21-30 y	63 (46)	164 (32)
31-50 y	67 (49)	233 (46)
51-70 y	21 (15)	110 (22)
>70 y	0	9 (2)
How long did your longest concussion symptoms last?^d^		
< 1 wk	21 (15)	14 (3)
1 wk to 1 mo	19 (14)	46 (9)
2 to 3 mo	13 (9)	29 (6)
4 to 12 mo	16 (12)	79 (15)
>1 y	69 (50)	339 (66)
Did not answer	0	4 (1)
Are your symptoms still ongoing?^d^		
Yes	77 (56)	414 (81)
No	44 (32)	84 (17)
Do not know or unsure	16 (12)	11 (2)
Did not answer	0	2 (0.4)
How did you sustain your concussion(s)?^d^		
Sports injury	83 (43)	225 (31)
Non–sports-related injury or fall	58 (30)	227 (31)
Motor vehicle collision	33 (17)	172 (24)
Physical abuse or assault	10 (5)	33 (5)
Other	11 (6)	66 (9)

Abbreviation: NA, not applicable.

^a^ Questions were optional; responses may not sum to the total number of respondents. Unless otherwise indicated, data are expressed as No. (%) of participants.

^b^ In survey 1, respondents could select more than 1 response. For survey 2, respondents could select only 1 response, which assigned them to the lived experience or clinician (health care professional) group.

^c^ Indicates not described in the categories listed.

^d^ Includes patients with concussion only: 138 for survey 1; 511 for survey 2.

A total of 1761 concussion questions and comments were collected from the 249 participants (Figure). Of these, 1515 (86%) were in scope and 246 (14%) were determined to be out of scope (eg, 1-word comments, no identifiable question, not concussion related). Of the 1515 questions that were in scope, 88 summary questions were generated.

**Figure.**
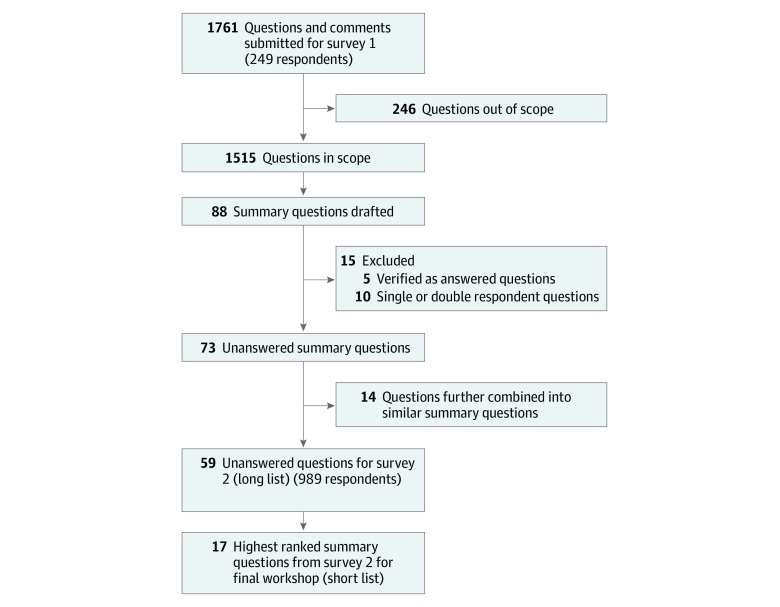
Flow Diagram of Summary Questions Across Survey 1 and Survey 2

### Evidence Verification

Of the 88 summary questions, 5 (6%) were determined to be sufficiently answered by existing research evidence (Box). This left 83 unanswered summary questions that were deemed by JLA methods and the steering group as too many to present in survey 2. The steering group pragmatically reduced the number of questions by further combining 14 questions into similar summary questions to create new summary questions, and by removing 9 questions submitted by only 1 respondent and 1 question submitted by only 2 respondents (eTable 1 in Supplement 1). Before removing these 10 questions, the steering group ensured that key issues of underrepresented groups were not lost. Therefore, 59 of 88 questions were carried forward to the interim prioritization survey (eTable 2 in Supplement 1).

Box. **List of the 5 Questions Considered Answered Based on Evidence Review**

- What is the effect of a delay in concussion diagnosis on symptom severity and outcomes?
- What relationship exists between neck injuries (eg, whiplash) and concussions?
- Are impact sensors (eg, helmet or mouthguard sensors) effective tools to help diagnose and assess concussions?
- What criteria are required for a health care provider to request a CT scan for a child with a head injury and possible concussion?
- What type of family support, education, and resources result in the best outcomes for families coping with concussion?

Abbreviation: CT, computed tomography.

### Interim Priority Setting: Survey 2

A total of 989 participants completed survey 2 (Table 1 and eFigure 2 in Supplement 1). Of these, 654 respondents (66%) had lived experience and 327 (33%) were clinicians. Eight respondents (1%) did not answer the question stating which type of participant they were. Their responses were not used to rank the unanswered questions. The mean (SD) age was 43.0 (4.2) years; 764 respondents (77%) identified as female, 206 (21%) as male, and 1 as other, and 18 preferred not to answer. Self-reported race and ethnicity or cultural background included Asian (53 [5%]), Black (11 [1%]), Indigenous (21 [2%]), Latino (12 [1%]), Middle Eastern (11 [1%]), and White (882 [89%]). Respondents were from across Canada except the 3 northern territories. Of the 511 patients with concussion, 226 (44%) had 3 or more concussions, 339 (66%) experienced concussion symptoms lasting longer than 1 year, and 414 (81%) reported ongoing concussion symptoms. Of all 723 injury mechanisms reported, 227 (31%) resulted from a fall not related to a sport and 225 (31%) were sports injuries.

Table 2 describes the top 21 unanswered research questions in concussion. The questions are sorted by combined rank and show separate rankings by groups with lived experience and clinicians. The steering group unanimously decided to select all questions that fell into either group’s top 12 ranked questions, which resulted in 17 questions selected for the final priority-setting workshop.

**Table 2.** The Combined Ranking of the Top 21 Questions by 981 Respondents of Survey 2^a^

Combined rank^b^	Summary question	Rank	
		Respondents with lived experience	Clinicians
1	What factors or tests best predict a prolonged recovery from concussion (known as postconcussion syndrome), and how can this information be used to develop a tailored strategy to manage the symptoms and support recovery?	4	2
2	What is the most effective way to manage headache associated with concussion, and should people with a prior diagnosis of migraine be treated differently to get the best outcomes?	6	1
3	How often does concussion result in cognitive impairment (eg, loss of attention, loss of memory, feeling foggy), and what is the best way to treat this?	2	8
= 4	What are the long-term effects of single or multiple concussions, and how do these effects impact day-to-day life?	1	12
= 4	How can the training of front-line physicians and other health care providers to recognize, diagnose, and manage concussion be improved and kept up to date?	7	6
6	What is the best way to determine the severity of a concussion, and how does the degree of severity affect the response to treatment and the time to fully recover?	5	9
7	What is the most effective way of differentiating prolonged concussion symptoms (known as postconcussion syndrome) from symptoms that are similar but unrelated to the concussion?	10	10
8	What is the effectiveness of early referral and treatment by a concussion specialist team (eg, with combined medical assessment, physical therapies, mental health support, and other rehabilitation services) on concussion outcomes and length of recovery?	17	7
= 9	What is the most effective test to track the progress of concussion treatment and monitor recovery over time?	21	4
= 9	What is the best way to assess and treat dizziness and balance problems after concussion?	14	11
11	What are the cumulative, long-term effects of multiple minor impacts to the head, or very mild concussions, on the brain and how does this affect day-to-day life?	8	19
12	After a concussion, what is the best approach for a return to physical activity, exercise, and sports (ie, timing, type, and intensity of activity) to give the best outcome?	25	3
= 13	What is the correlation between concussion severity and disturbed sleep patterns? What tools, aids, medications, or other interventions are most effective in managing these sleep disturbances?	11	21
= 13	How do preexisting mental health conditions (eg, anxiety, depression, stress) influence the severity of concussion symptoms and time to recovery?	12	20
15	What structural and/or functional (eg, molecular, biochemical) changes occur in the brain with a concussion? How does the intensity and direction of forces involved in the head trauma cause the brain injury, and does location of the brain injury impact symptoms and severity?	3	30
16	After a concussion, what is the best timing and approach to return to cognitive activities in educational settings (eg, school or university) and work settings to give the best outcome?	30	5
= 17	What is the role of therapeutic glasses and vision therapy (eg, eye movement and focusing exercises) in managing concussion symptoms?	21	17
= 17	Are there medications that can improve concussion recovery?	19	19
19	When is a neuropsychological assessment (ie, testing of cognition) most effective for treatment planning after concussion, and in what circumstances is this type of assessment recommended?	28	14
= 20	How often do concussions result in long-term cognitive decline such as dementia and/or chronic traumatic encephalopathy (CTE)? (CTE is a progressive brain condition that may occur after repeated blows to the head.) How can these long-term effects be best prevented or treated?	9	34
= 20	What is the effectiveness of neck and core muscle strengthening exercises in preventing or reducing the severity of a concussion?	27	16

^a^ Eight of the 989 respondents did not record the type of participant and were excluded from the ranking process.

^b^ To ensure equal influence for respondents with lived experience and clinicians, questions were ranked from highest to lowest for each group and the 2 scores were summed to give a total score which determined the combined rank order for each question. The equals sign denotes equal rank.

### Final Priority-Setting Workshop

Twenty-four participants (13 with lived experience and 11 clinicians) attended the workshop. Of the 13 with lived experience, 8 (62%) identified as female and 5 (38%) as male. One participant was younger than 18 years, and the mechanism of concussion injury included sports (6 [46%]), non–sport-related falls (4 [31%]), motor vehicle–related injuries (2 [15%]), and assault (1 [8%]). Clinicians included 2 physiotherapists, 2 neuropsychologists, 1 nurse, 1 athletic therapist, and 5 physicians (pediatric emergency, psychiatry, rehabilitation, and sports medicine). Consensus was reached on the final ranking of the top 10 unanswered research questions in concussion (Table 3).

**Table 3.** List of the Top 10 Unanswered Research Questions Focused on Concussion

Rank	Question
1	What factors or tests best predict a prolonged recovery from concussion (known as postconcussion syndrome), and how can this information be used to develop a tailored strategy to manage the symptoms and support recovery?
2	After a concussion, what is the best timing and approach to return to cognitive activities in educational settings (eg, school or university) and work settings to give the best outcome?
3	What is the effectiveness of early referral and treatment by a concussion specialist team (eg, with combined medical assessment, physical therapies, mental health support, and other rehabilitation services) on concussion outcomes and length of recovery?
4	After a concussion, what is the best approach for a return to physical activity, exercise, and sports (ie, timing, type, and intensity of activity) to give the best outcome?
5	What is the most effective way to manage headache associated with concussion, and should people with a prior diagnosis of migraine be treated differently to get the best outcomes?
6	What is the most effective way of differentiating prolonged concussion symptoms (known as postconcussion syndrome) from symptoms that are similar but unrelated to the concussion?
7	What structural and/or functional (eg, molecular, biochemical) changes occur in the brain with a concussion? How do the intensity and direction of forces involved in the head trauma cause the brain injury, and does location of the brain injury impact symptoms and severity?
8	What are the long-term effects of single or multiple concussions, and how do these effects impact day-to-day life?
9	What is the correlation between concussion severity and disturbed sleep patterns? What tools, aids, medications, or other interventions are most effective in managing these sleep disturbances?
10	How can the training of front-line physicians and other health care providers to recognize, diagnose, and manage concussion be improved and kept up to date?

A postworkshop anonymous survey was completed by all 24 attendees. Overall, all attendees (100%) strongly agreed or agreed that they were able to share thoughts and opinions effectively, 23 (96%) strongly agreed or agreed that the facilitators were fair and impartial, and 22 (92%) strongly agreed or agreed that the process of determining the top 10 questions was fair and robust.

## Discussion

This JLA PSP gathered opinions from more than 1000 patients, caregivers, and clinicians and, through a multistep process, identified the top 10 unanswered research questions that matter most to those affected by concussion. The project brought together a broad and diverse group of patients, caregivers, and clinicians from across Canada and, to our knowledge, is the first patient-oriented research priority-setting project in concussion. The top 10 questions identify priority areas on which researchers and research networks can focus to improve outcomes for people living with concussion.

Taken as a whole, the list of research priorities is broad in scope and reflects the diverse concerns of patients, caregivers, and clinicians. Priorities include early and accurate diagnosis after a head injury, specific symptom management, approaches to ensure a safe return to physical and cognitive activities after concussion, predicting and preventing prolonged symptoms and poor outcomes, and understanding the pathophysiology of concussion.

The top-ranked question focuses on identifying patients at high risk for prolonged symptoms at the time of diagnosis and developing a personalized management strategy. With up to 30% of people with concussion experiencing symptoms longer than 1 month,^4,5,6^ early and accurate identification of risk factors for prolonged symptoms was deemed the highest priority. Risk factors associated with prolonged recovery have been identified, and a clinical risk score has been validated for pediatric patients^6,14^ and is now in development for adult patients.^27^ However, despite considerable research, there are no validated physiological, serological, or radiological biomarkers that can accurately diagnose concussion and predict recovery.^28,29,30,31^ Therefore, more research is needed to implement existing validated tools into clinical pathways and to develop additional biomarkers and strategies to refine diagnosis and personalize therapies to improve outcomes.

Three questions focused on improving early diagnosis and management of concussion. The first concerns the role of interdisciplinary teams of health care professionals (eg, physicians, physiotherapists, neuropsychologists, occupational therapists, mental health professionals, vision specialists) in assessing and managing concussion and asks which patients should be referred early to these teams to achieve the best outcomes. A second question focused on the need to develop approaches to differentiate symptoms due to concussion from those of nonconcussion medical problems that may have similar symptoms but require different therapies. The third question concerns the variation in abilities of clinicians to recognize and manage concussion. With concussion diagnosis and management recommendations changing frequently, educating clinicians on concussion was considered important to improve recognition and standardize management.

Two highly ranked questions focused on identifying the best approach for a safe and effective return to activities. Recent evidence has shown that after a short period of initial rest (24-48 hours), mild to moderate physical and cognitive activity is beneficial for recovery.^14,32^ Some patients have difficulty increasing mental and physical activity without exacerbating postconcussion symptoms. Effective strategies to reintroduce cognitive and physical activities in those with refractory symptoms, to reduce time away from school, work, and/or physical activity, and to improve recovery time are needed. Two additional management-related questions focused on the effectiveness of interventions to manage concussion-related chronic headache and sleep disturbances.

Five questions were deemed to be sufficiently answered by literature review. These answered questions highlight gaps in knowledge translation and should be a priority for dissemination to stakeholders and the public to improve awareness and enhance implementation of existing research evidence.

### Strengths and Limitations

Strengths of this study include the methodological rigor and transparent nature of the JLA-PSP methods. The JLA PSP is a widely used and highly cited approach for facilitating collaboration between patients and clinicians to prioritize research questions.^20,22^ Results were analyzed transparently and are shared openly on the project website. Other strengths include a mix of ages of survey participants across Canada, including speakers of both official languages and those in urban and rural areas. The steering group, survey respondents, and workshop participants included a diverse group of clinicians who treat people with concussion, allowing for different perspectives across professions.

This study has some limitations. First, it was challenging to recruit people from some racial, ethnic, and cultural backgrounds, particularly those who identify as Black, Latino, and Middle Eastern. Our study was able to recruit 4% of individuals with concussion who self-identified as Indigenous (5% of Canadians identify as being Indigenous).^33^ Second, we may have had difficulty reaching groups who do not have internet access. However, both a paper version of the survey and telephone completion were offered. Third, greater than 50% of survey participants had concussion symptoms lasting longer than 1 year, and this may have resulted in research questions reflecting more chronic concerns. Despite this limitation, many questions in the top 10 focused on acute concussion management. Fourth, as the surveys were completely anonymous, we were not able to determine whether people completed them more than once. Fifth, although many questions on concussion prevention came from survey 1, they were not highly ranked by participants in survey 2 and therefore not selected as priorities to include in the final workshop. We believe that this omission does not reflect a lack of importance of research on concussion prevention. Rather, we believe it reflects the overwhelming importance of diagnosis, treatment, and prognosis as the major concerns of individuals living with concussion and clinicians managing concussion. Finally, this is a Canadian study, and the research priorities may not reflect people from countries with different health care expertise, access, and resourcing.

## Conclusions

This PSP of patients, caregivers, and clinicians worked together in an equal partnership to identify the top 10 patient-oriented research questions in concussion. These priority questions help inform the concussion research agenda and may be useful to stakeholders, researchers, clinicians, funders, hospital leaders, and policy makers. The goal is that these questions will stimulate funding for concussion research that will answer questions that matter most to those living with concussion and those who care for them.

## Notes

Supplement 1. **eFigure 1.** Survey 1 Used in Concussion Priority-Setting Partnership **eFigure 2.** Survey 2 Used in Concussion Priority-Setting Partnership **eTable 1.** List of Unanswered Questions Submitted by 1 or 2 Respondents (n = 10) **eTable 2.** Long List of Unanswered Summary Questions in Concussion (n = 59)

Supplement 2. **Nonauthor Collaborators.** Canadian Traumatic Brain Injury Research Consortium

Supplement 3.
Data Sharing Statement
