# Supplementary material for: Partnering With Patients, Caregivers, and Clinicians to Determine Research Priorities for Concussion
Source: JAMA Netw Open. 2023 Jun 7;6(6):e2316383. doi: 10.1001/jamanetworkopen.2023.16383 (PMC10248744; doi:10.1001/jamanetworkopen.2023.16383)
Supplement: Supplement 1. — eFigure 1. Survey 1 Used in Concussion Priority-Setting Partnership eFigure 2. Survey 2 Used in Concussion Priority-Setting Partnership eTable 1. List of Unanswered Questions Submitted by 1 or 2 Respondents (n = 10) eTable 2. Long List of Unanswered Summary Questions in Concussion (n = 59) [file jamanetwopen-e2316383-s001.pdf]

Supplementary Online Content

Osmond MH, Legace E, Gill PJ, et al; Canadian Traumatic Brain Injury Research Consortium. Partnering with patients, caregivers, and clinicians to determine research priorities for concussion. *JAMA Netw Open*. 2023;6(6):e2316383. doi:10.1001/jamanetworkopen.2023.16383

eFigure 1. Survey 1 Used in Concussion Priority-Setting Partnership

eFigure 2. Survey 2 Used in Concussion Priority-Setting Partnership

eTable 1. List of Unanswered Questions Submitted by 1 or 2 Respondents (n = 10)

eTable 2. Long List of Unanswered Summary Questions in Concussion (n = 59)

This supplementary material has been provided by the authors to give readers additional information about their work.

© 2023 Osmond MH et al. *JAMA Network Open.*

eFigure 1. Survey 1 Used in Concussion Priority-Setting Partnership

Confidential

Concussion Priority Setting Partnership Survey

We need your voice to shape the future of concussion research

Do you have any questions about the diagnosis, prevention, treatment or care of concussion which you feel could benefit from research.

Please tell us what you think.

You are being invited to participate in a research study to help determine the top 10 questions that concussion researchers should answer.

We are a team of healthcare providers, patients, caregivers and researchers who are working together to identify research priorities in concussion. We want thoughts and ideas from people across Canada with different experiences and backgrounds.

If you are (or have been):

A person who has had one or multiple concussions A family member or caregiver of a person living with a concussion A healthcare provider who takes care of people with concussion A coach, teacher, colleague or friend of a person with concussion We want to hear from you!

Your questions will help us guide future research to identify solutions and improve the lives for people with concussion.

Information about the survey

Your participation is completely voluntary, and the survey involves minimal risk to you. The benefits, however, may impact society by helping increase knowledge about concussion. You do not have to answer any questions that you do not want to answer for any reason. You are free to withdraw from the study at any time. Questions you enter into this anonymous survey will be shared but will not be linked to you personally in any way. This means once you have submitted your survey, your responses cannot be withdrawn from the study. Survey data will be stored in a secure location at CHEO. Data will be kept for 7 years and then destroyed. In the future, you may be invited to participate in a second phase of this study which will ask you to rank the research questions most important to you.

This is a brief 5 question survey which may take about 15 minutes to complete.

By completing this survey, you are agreeing to participate in this study.
The CHEO Research Ethics Board (REB) has reviewed and approved this study.

To complete this survey in English, click the "Next Page" button below.
To complete this survey in French, click here.

If you need more information or need help to complete the survey, please contact us by emailing (xxx) or by telephone at (xxx) xxx-xxxx ext.xxxx

projectredcap.org REDCap®

CONCUSSION PRIORITY SETTING PARTNERSHIP

© 2023 Osmond MH et al. *JAMA Network Open.*

Section1: Your Questions

What questions about concussion would you most like answered by research?

Think of your own personal or professional experience with concussion.

Do you have any questions about how concussion is diagnosed, treated, prevented and managed? Are there any uncertainties about patient concussion care or areas where you feel there should be more evidence to guide care? We're looking for questions about any part of the concussion path, from diagnosis to treatment to control of symptoms to prevention. These questions can be as specific as individual therapies, or as broad as healthcare policies.

Please share your questions with us! There are no right or wrong answers and there is no need to check if your questions have already been answered by research.

To help guide you, there are 5 questions below that ask if you have questions regarding diagnosis, treatment, managing symptoms, prevention and prognosis of concussion.

(Please note: The 5 questions are just a guide - there is no need to provide a response for all 5 questions.)

Please list your questions in the boxes below that you would most like answered by research.

1. What questions about the diagnosis of concussion would you like answered by research? Please list them here.

(Diagnosis includes the detection, examining, testing and confirming of concussion.)

____

2. What questions about the treatment of concussion would you like answered by research? Please list them here.

(Treatment includes the use of specific therapies including medications, occupational therapy, physiotherapy, rest and exercise protocols, complementary medicine, etc.)

____

3. What questions about managing symptoms and day-to-day life with concussion would you like answered by research? Please list them here.

(These include any modifications or adjustments to everyday life, including life at work or school, emotional health, relationships, leisure activities, sleep, etc.)

____

4. What questions about the prevention of concussion would you like answered by research? Please list them here.

(Prevention is the effort, where possible, to stop concussions from happening or reduce the seriousness of a concussion.)

____

© 2023 Osmond MH et al. *JAMA Network Open.*

5. What questions about the prognosis of concussion would you like answered by research? Please list them here.

(Prognosis is the prediction of how a concussion will impact one's life and how long it may take to feel well again. For example: how bad one's symptoms are, how long one's symptoms will last etc.)

The next page asks some questions about your background. These questions will ensure we are reaching a wide range of people across Canada.

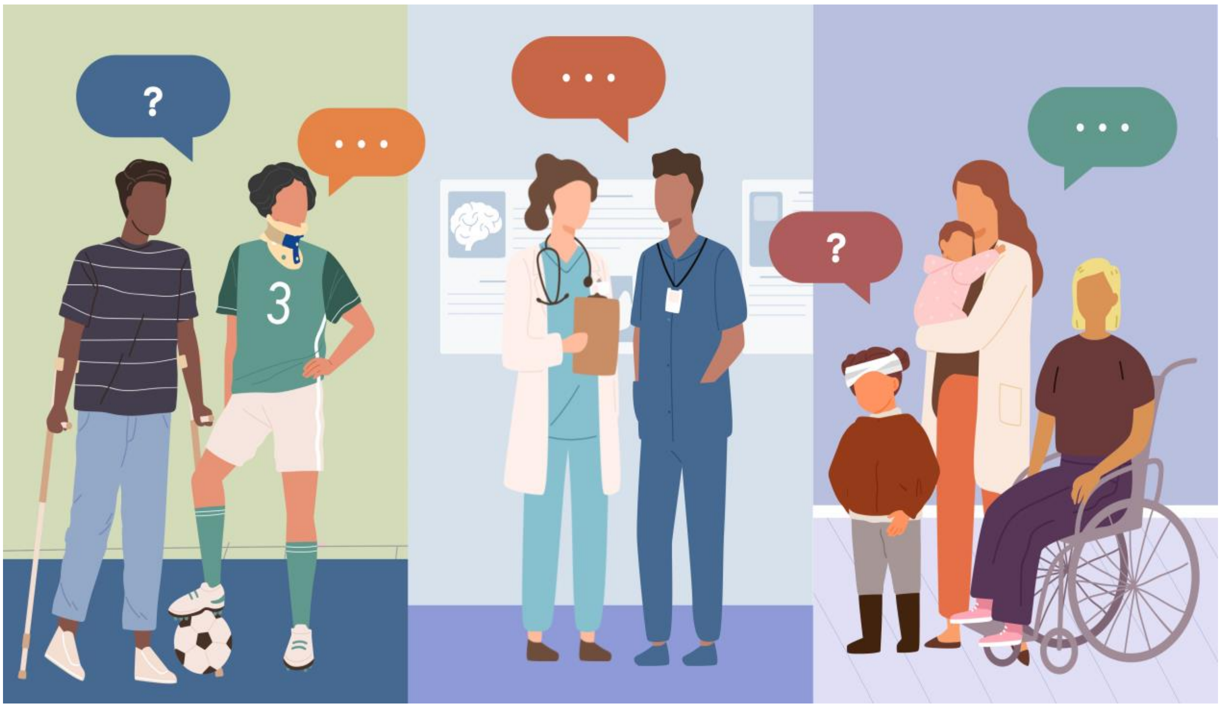

If you have any questions or need help to complete the survey please contact us by emailing (xxx) or by telephone at (xxx) xxx-xxxx ext.xxxx

© 2023 Osmond MH et al. *JAMA Network Open.*

Section 2: Tell us About Yourself!

1.	Which of the following best describes you? (choose all that apply)	◯ Person who has or has had a concussion<br>◯ Parent or family member of a person with a concussion<br>◯ Caregiver/ former caregiver of a person with a concussion<br>◯ Friend or colleague of a person with a concussion<br>◯ Healthcare professional<br>◯ Teacher<br>◯ Coach<br>◯ Member of an organization representing people with concussion
a.	How many concussions have you had?	◯ 1<br>◯ 2<br>◯ 3 or more<br>◯ Don't know/unsure
b.	At which age(s) did you have your concussion(s)? (check all that apply)	☐ 0-5 years<br>☐ 6-10 years<br>☐ 11-15 years<br>☐ 16-20 years<br>☐ 21-30 years<br>☐ 31-50 years<br>☐ 51-70 years<br>☐ Greater than 70 years
c.	How long did your longest concussion symptoms last?	◯ Less than 1 week<br>◯ 1 week to 1 month<br>◯ 2 to 3 months<br>◯ 4 to 12 months<br>◯ Greater than 1 year
d.	Are your symptoms still ongoing?	◯ Yes<br>◯ No
e.	How did you sustain your concussion(s)? (choose all that apply)	☐ Sports injury<br>☐ Non-sports related injury or fall<br>☐ Motor vehicle collision<br>☐ Physical abuse or assault<br>☐ Other
i.	What is your specific healthcare profession?	◯ Physician<br>◯ Nurse<br>◯ Physiotherapist<br>◯ Occupational Therapist<br>◯ Social Worker<br>◯ Neuropsychologist<br>◯ Mental Health<br>◯ Practitioner Chiropractor<br>◯ Athletic Therapist<br>◯ Other
2.	What is your current age in years?	______________________

© 2023 Osmond MH et al. *JAMA Network Open.*

3.	How do you identify?	◯ Female<br>◯ Male<br>◯ Other<br>◯ Prefer not to say
4.	What racial background best describes you?<br><br>Check all that apply.	☐ Black (African, Afro-Caribbean, African Canadian descent)<br>☐ East/Southeast Asian (e.g. Chinese, Korean, Japanese, Vietnamese, Thai, other Southeast Asian descent)<br>☐ Indigenous (First Nations, Métis, Inuk/Inuit)<br>☐ Latino (Latin American, Hispanic descent)<br>☐ Middle Eastern (Arab, Persian, West Asian descent)<br>☐ South Asian (e.g., East Indian, Pakistani, Bangladeshi)<br>☐ White (North American or European descent)<br>☐ Prefer not to say<br>☐ Other
5.	What is the language spoken in your home?	◯ English<br>◯ French<br>◯ Other<br>◯ Prefer not to say
6.	Where do you reside?	◯ Canada<br>◯ Other
		______________________
a.	What province or territory do you live in?	◯ Alberta<br>◯ British Columbia<br>◯ Manitoba<br>◯ New Brunswick<br>◯ Nova Scotia<br>◯ Northwest Territories<br>◯ Newfoundland and Labrador<br>◯ Nunavut<br>◯ Ontario<br>◯ Prince Edward Island<br>◯ Quebec<br>◯ Saskatchewan<br>◯ Yukon
7.	What best describes where you live?	◯ Urban<br>◯ Rural

If you have any questions or need help to complete the survey, please contact us by telephone (xxx-xxx-xxxx) or email (xxx or visit our website.

Please click the "Submit" button below to submit your survey!

projectredcap.org

© 2023 Osmond MH et al. *JAMA Network Open.*

eFigure 2. Survey 2 Used in Concussion Priority-Setting Partnership

Confidential

Concussion Priority Setting Top 10 Survey

Choose your priorities for the future of research in concussion! You are being invited to participate in a research study to help determine the top 10 questions that concussion researchers should answer.

This survey will take 10 - 15 minutes to complete.

We are a team of people living with concussion, caregivers, and healthcare providers who are working together to identify research priorities in concussion.

If you are (or have been):

A person who has had one or multiple concussions. A family member or caregiver of a person living with a concussion. A healthcare provider who takes care of people with concussion. A coach, teacher, or friend of a person with concussion. We want to hear from you!

Information About the Survey

What is the survey for?

We recently conducted a survey that asked people to submit their unanswered questions about concussion. We want to use this information to guide future research and make sure researchers focus on the most urgent needs of people living with concussion.

249 Canadians submitted 1,761 questions about the diagnosis, treatment, management, prevention, and prognosis of concussion. Our team reviewed each submission, summarized them into questions and removed any that have already been answered by previous research.

Now, in this second survey, we need to know which ones are most important to you!

Who should take part in this second survey?
This survey is open to people who have had one or multiple concussions, family members or caregivers of people living with concussion, healthcare providers who take care of people with concussion, and coaches, teachers, and/or friends of people with concussion.

Your participation is completely voluntary, and the survey involves minimal risk to you. The benefits, however, may impact society by helping increase knowledge about concussion. You do not have to answer any questions that you do not want to answer for any reason. You are free to withdraw from the study at any time. Answers you enter into this anonymous survey will be shared but will not be linked to you personally in any way. This means once you have submitted your survey, your responses cannot be withdrawn from the study. Survey data will be stored in a secure location at CHEO, the children's hospital in Ottawa. Data will be kept for 7 years and then destroyed. By completing this survey, you are agreeing to participate in this study. The CHEO Research Ethics Board (REB) has reviewed and approved this study.

To complete this survey in English, click the "Next Page" button below.

To complete this survey in French, click here.

If you need more information or need help to complete the survey, please contact us by emailing (xxx) or by telephone at (xxx) xxx-xxxx ext.xxxx

CONCUSSION PRIORITY SETTING PARTNERSHIP

projectredcap.org

REDCap®

© 2023 Osmond MH et al. *JAMA Network Open.*

Section I: Choose Questions that are Important to You!

As you will see, we have collected a lot of questions (59 in total). This first step is just to help you narrow them down.

Please have a look through this list and check off any you feel are really important to you. You can choose as many as you like but try not to check them all!

After, in Section 2, you'll have the chance to pick your Top 10! **Questions 1 - 10**

☐ What are the cumulative, long-term effects of multiple minor impacts to the head, or very mild concussions, on the brain and how does this effect day-to-day life?

☐ What is the best way to assess and treat dizziness and balance problems after concussion?

☐ What types of physical and cognitive rest have the most positive impact on recovery from concussion, and what is the optimal duration of the rest? What specific activities worsen concussion symptoms and should be avoided?

☐ What are the key strategies to ensure a safe, effective, and productive return to work for people recovering from a concussion?

☐ What is the most effective way of differentiating prolonged concussion symptoms (known as post-concussion syndrome) from symptoms that are similar but unrelated to the concussion?

☐ To what extent does the age at which a concussion occurs (i.e., child, adolescent, adult, older adult) impact the severity of symptoms and time to recover? Should treatment and prevention strategies be tailored to age to be most effective?

☐ What is the role of physical examination tests (e.g., eye movement, balance and coordination tests) in accurately and objectively diagnosing a concussion and predicting its severity?

☐ How effective are cannabis products (cannabinoids such as CBD) in treating specific symptoms of concussion?

☐ What is the effectiveness of early referral and treatment by a concussion specialist team (with combined medical assessment, physical therapies, and mental health support) on concussion outcomes and length of recovery?

☐ How common are hearing problems (i.e., sound hypersensitivity or tinnitus (ringing in the ears)) after concussion and how are these best treated?

© 2023 Osmond MH et al. *JAMA Network Open.*

Choose the Questions that are Most Important to You

Questions 11 - 20

☐ How important is proper nutrition in managing concussion symptoms and are there benefits to taking nutritional supplements (e.g., vitamins)?

☐ What is the effectiveness of neck and core muscle strengthening exercises in preventing or reducing the severity of a concussion?

☐ What methods should be used to diagnose concussion in those who may not be able to clearly communicate their symptoms? (e.g.; young children and older adults with cognitive decline).

☐ Do concussions cause attention deficit hyperactivity disorder (ADHD)?

☐ How effective is brain imaging (e.g., MRI) in confirming concussion, evaluating concussion severity, and predicting recovery from concussion?

☐ How does sex and gender affect concussion susceptibility, severity of symptoms, and time to recover? Should treatment and prevention strategies be tailored to sex and gender to be most effective?

☐ What is the most effective way to accurately identify and safely manage a suspected sports concussion at the time of injury?

☐ How often does concussion result in cognitive impairment (i.e., loss of attention, loss of memory, feeling foggy), and what is the best way to treat this?

☐ What protective equipment (e.g., helmets, face masks, head gear, mouth guards) is most effective in preventing concussion in sports?

☐ How often do concussions result in dementia and/or Chronic Traumatic Encephalopathy (CTE)? (CTE is a progressive brain condition that occurs after repeated blows to the head). How can these long-term effects be best prevented or treated?

projectredcap.org REDCap

© 2023 Osmond MH et al. *JAMA Network Open.*

Choose the Questions that are Most Important to You

Questions 21 - 30

- [ ] How and when should a person be assessed to determine whether it is safe for them to return to driving after concussion?

- [ ] How effective is acupuncture in treating concussion symptoms?

- [ ] How do hormones affect the symptoms and severity of a concussion, and how does a concussion affect the function of hormones?

- [ ] What is the role of therapeutic glasses and vision therapy (e.g., eye movement and focusing exercises) in managing concussion symptoms?

- [ ] What is the best way to reduce the number of concussions suffered by students while at school? What is the most effective way to train school personnel to recognize symptoms of concussion and provide guidance to students with concussion?

- [ ] How effective are physical manipulation therapies, performed by physiotherapists, osteopathic physicians and/or chiropractors, in the treatment of concussion?

- [ ] After a concussion, what is the best timing and approach to return to learning activities and educational settings (e.g., school or university) to give the best outcome?

- [ ] What is the correlation between concussion severity and disturbed sleep patterns? What tools, aids, medications, or other interventions are most effective in managing these sleep disturbances?

- [ ] What is the best way to manage light sensitivity caused by a concussion?

- [ ] What is the effect of socioeconomic and cultural factors (e.g. race, income, sexual orientation, language, housing, social/community connection) on the diagnosis, management and prognosis of concussion?

projectredcap.org REDCap®

© 2023 Osmond MH et al. *JAMA Network Open.*

Choose the Questions that are Most Important to You

Questions 31 - 40

☐ What factors or tests best predict a prolonged recovery from concussion (known as post-concussion syndrome), and how can this information be used to develop a tailored strategy to manage the symptoms and support recovery?

☐ What is the effectiveness of meditation and other mindfulness practices on concussion recovery?

☐ What is the effectiveness of limiting screen time (e.g., computer monitors, video games, smart phones) in reducing symptoms after a concussion? What is the best approach for managing screen time?

☐ Should treatment of concussion differ between athletes and non-athletes to ensure the most effective recovery, and if so, what is the best treatment for each group?

☐ When is a neuropsychological assessment (i.e., testing of cognition) most effective for treatment planning after concussion, and in what circumstances is this type of assessment recommended?

☐ Does the process of making an insurance claim, or filing a lawsuit, related to a concussion injury worsen or prolong concussion symptoms?

☐ How does the relationship between parent and child impact symptoms and time to recovery for children who sustain a concussion?

☐ What game/competition and practice modifications (e.g., rule changes, behavioural interventions, revised training techniques) are most effective in preventing concussion in sports?

☐ What is the best way to determine the severity of a concussion, and how does the degree of severity affect the response to treatment and the time to fully recover?

☐ How can a concussion due to intimate partner violence best be detected and treated to improve long-term day-to-day living?

projectredcap.org REDCap®

© 2023 Osmond MH et al. *JAMA Network Open.*

Choose the Questions that are Most Important to You

Questions 41 - 50

☐ Are there medications that can improve concussion recovery?

☐ What is the best way to diagnose concussion in the emergency department?

☐ How can the training of front-line doctors and other healthcare providers to recognize, diagnose and manage concussion be improved and kept up to date?

☐ What is the most effective way to reduce the number of concussions occurring in a workplace?

☐ What is the most effective tool (e.g., website, app, paper-based instructions) to help people with concussion adjust their day-to-day activities to manage symptoms?

☐ After a concussion, what is the best approach for a return to physical activity, exercise, and sports (i.e., timing, type, and intensity of activity) to give the best outcome?

☐ At what age should full body contact be introduced in contact sports and what strategies should be implemented to prevent concussions caused by full body contact?

☐ What genetic factors influence the risk of concussion, prolonged symptoms, and long-term effects of concussion?

☐ What are the long-term effects of single or multiple concussions, and how do these effects impact day-to-day life?

☐ How does sharing information with a concussion patient about their prognosis affect the patient's symptoms and outcomes?

© 2023 Osmond MH et al. *JAMA Network Open.*

Choose the Questions that are Most Important to You

Questions 51 - 59

☐ Do concussion outcomes improve by increasing public awareness of: 1) the signs and symptoms of a concussion; 2) the seriousness of a concussion; and/or, 3) when and where to seek care following a concussion?

☐ What is the role of biological tests (e.g., blood or saliva tests) in accurately and objectively diagnosing a concussion and predicting its severity?

☐ What is the optimal type and timing of mental health support in the treatment of concussion?

☐ How do pre-existing mental health conditions (e.g., anxiety, depression, stress) influence the severity of concussion symptoms and time to recovery?

☐ What structural changes occur in the brain with a concussion? How does the intensity and direction of forces involved in the head trauma cause the brain injury, and does location of the brain injury impact symptoms and severity?

☐ What is the most effective way to manage headache associated with concussion, and should people with a prior diagnosis of migraine be treated differently to get the best outcomes?

☐ Which activities or factors place people at highest risk for getting a concussion and how can these be modified or taken into consideration to reduce the risk of concussion?

☐ After the first concussion, what is the risk of getting future concussions and are they more severe?

☐ What is the most effective test to track the progress of concussion treatment and monitor recovery over time?

© 2023 Osmond MH et al. *JAMA Network Open.*

Section 2: Now Pick your Top 10!

Shown below are the questions you selected in Section 1. Please choose up to 10 which you want to go through to help us find the overall Top 10.

Count

____

You have selected [count] questions

You have selected [count] questions

You have selected [count] questions

In the next page, we will also ask a few questions about you. This is so we can make sure that a wide range of people take part.

projectredcap.org REDCap®

© 2023 Osmond MH et al. *JAMA Network Open.*

Section 3: Tell us About Yourself!

1.	Which of the following best describes you? (please select only one)	◯ Person who has or has had a concussion<br>◯ Parent or family member of a person with a concussion<br>◯ Caregiver/ former caregiver of a person with a concussion<br>◯ Friend or colleague of a person with a concussion<br>◯ Healthcare professional<br>◯ Teacher<br>◯ Coach<br>◯ Member of an organization representing people with concussion
a.	How many concussions have you had?	◯ 1<br>◯ 2<br>◯ 3 or more<br>◯ Don't know/unsure
b.	At which age(s) did you have your concussion(s)? (check all that apply)	☐ 0-5 years<br>☐ 6-10 years<br>☐ 11-15 years<br>☐ 16-20 years<br>☐ 21-30 years<br>☐ 31-50 years<br>☐ 51-70 years<br>☐ Greater than 70 years
c.	How long did your longest concussion symptoms last?	◯ Less than 1 week<br>◯ 1 week to 1 month<br>◯ 2 to 3 months<br>◯ 4 to 12 months<br>◯ Greater than 1 year
d.	Are your symptoms still ongoing?	◯ Yes<br>◯ No
e.	How did you sustain your concussion(s)? (choose all that apply)	☐ Sports injury<br>☐ Non-sports related injury or fall<br>☐ Motor vehicle collision<br>☐ Physical abuse or assault<br>☐ Other
i.	What is your specific healthcare profession?	◯ Physician<br>◯ Nurse<br>◯ Physiotherapist<br>◯ Occupational Therapist<br>◯ Social Worker<br>◯ Neuropsychologist<br>◯ Mental Health<br>◯ Practitioner Chiropractor<br>◯ Athletic Therapist<br>◯ Other
2.	What is your current age in years?	____________________

© 2023 Osmond MH et al. *JAMA Network Open.*

3.	How do you identify?	◯ Female<br>◯ Male<br>◯ Other<br>◯ Prefer not to say
4.	What racial background best describes you?<br><br>Check all that apply.	☐ Black (African, Afro-Caribbean, African Canadian descent)<br>☐ East/Southeast Asian (e.g. Chinese, Korean, Japanese, Vietnamese, Thai, other Southeast Asian descent)<br>☐ Indigenous (First Nations, Métis, Inuk/Inuit)<br>☐ Latino (Latin American, Hispanic descent)<br>☐ Middle Eastern (Arab, Persian, West Asian descent)<br>☐ South Asian (e.g., East Indian, Pakistani, Bangladeshi)<br>☐ White (North American or European descent)<br>☐ Prefer not to say<br>☐ Other
5.	What is the language spoken in your home?	◯ English<br>◯ French<br>◯ Other<br>◯ Prefer not to say
6.	Where do you reside?	◯ Canada<br>◯ Other
		__________________
a.	What province or territory do you live in?	◯ Alberta<br>◯ British Columbia<br>◯ Manitoba<br>◯ New Brunswick<br>◯ Nova Scotia<br>◯ Northwest Territories<br>◯ Newfoundland and Labrador<br>◯ Nunavut<br>◯ Ontario<br>◯ Prince Edward Island<br>◯ Quebec<br>◯ Saskatchewan<br>◯ Yukon
7.	What best describes where you live?	◯ Urban<br>◯ Rural

If you have any questions or need help to complete the survey, please contact us by telephone (xxx-xxx-xxxx) or email (xxx) or visit our website.

Please click the "Submit" button below to submit your survey!

projectredcap.org REDCap®

© 2023 Osmond MH et al. *JAMA Network Open.*

eTable 1. List of Unanswered Questions Submitted by 1 or 2 Respondents (n = 10)

1. What prevents athletes from self-reporting possible concussion symptoms and how can self-reporting be improved?
2. How can the severity of concussion resulting from motor vehicle accidents be reduced?
3. Do aspects of the interactions between the patient and health care provider (e.g., restricted time in appointment, judging the patient) affect concussion recovery?
4. How effective is transcranial stimulation (i.e., using magnetic fields to stimulate nerve cells in the brain) for treating concussion?
5. How effective are designated “concussion spotters” in recognizing concussion in professional sports?
6. Are devices which detect abnormalities in brain electrical activity (e.g., EEG) effective tools in diagnosing and evaluating a concussion?
7. What is the role of physical exertion testing (i.e., monitoring the body’s response to exercise) in assessing concussion severity and assessing readiness to return to sport or physical activity?
8. What are the best practices for evaluating and treating autonomic dysregulation (i.e., a condition where there in imbalance of the nervous system that works unconsciously to regulate body processes) that can occur with concussion?
9. Do psychological traits of the patient (e.g., fears, shame/embarrassment, inability to describe symptoms, focusing excessively on symptoms) affect concussion recovery?
10. What are the similarities and differences between athlete and non-athlete populations for concussion diagnosis, treatment/management, and prognosis?

© 2023 Osmond MH et al. *JAMA Network Open.*

eTable 2. Long List of Unanswered Summary Questions in Concussion (n = 59)

1. What is the role of biological tests (e.g., blood or saliva tests) in accurately and objectively diagnosing a concussion and predicting its severity?

2. What is the optimal type and timing of mental health support in the treatment of concussion?

3. How do pre-existing mental health conditions (e.g., anxiety, depression, stress) influence the severity of concussion symptoms and time to recovery?

4. What structural changes occur in the brain with a concussion? How does the intensity and direction of forces involved in the head trauma cause the brain injury, and does location of the brain injury impact symptoms and severity?

5. What is the most effective way to manage headache associated with concussion, and should people with a prior diagnosis of migraine be treated differently to get the best outcomes?

6. Which activities or factors place people at highest risk for getting a concussion and how can these be modified or taken into consideration to reduce the risk of concussion?

7. After the first concussion, what is the risk of getting future concussions and are they more severe?

8. What is the most effective test to track the progress of concussion treatment and monitor recovery over time?

9. How do hormones affect the symptoms and severity of a concussion, and how does a concussion affect the function of hormones?

10. What is the role of therapeutic glasses and vision therapy (e.g., eye movement and focusing exercises) in managing concussion symptoms?

11. What is the effectiveness of limiting screen time (e.g., computer monitors, video games, smart phones) in reducing symptoms after a concussion? What is the best approach for managing screen time?

12. Should treatment of concussion differ between athletes and non-athletes to ensure the most effective recovery, and if so, what is the best treatment for each group?

13. When is a neuropsychological assessment (i.e., testing of cognition) most effective for treatment planning after concussion, and in what circumstances is this type of assessment recommended?

14. Does the process of making an insurance claim, or filing a lawsuit, related to a concussion injury worsen or prolong concussion symptoms?

15. How does the relationship between parent and child impact symptoms and time to recovery for children who sustain a concussion?

16. What game/competition and practice modifications (e.g., rule changes, behavioral interventions, revised training techniques) are most effective in preventing concussion in sports?

17. What is the best way to determine the severity of a concussion, and how does the degree of severity affect the response to treatment and the time to fully recover?

18. How can a concussion due to intimate partner violence best be detected and treated to improve long-term day-to-day living?

19. How and when should a person be assessed to determine whether it is safe for them to return to driving after concussion?

20. What is the effectiveness of neck and core muscle strengthening exercises in preventing or reducing the severity of a concussion?

21. What methods should be used to diagnose concussion in those who may not be able to clearly communicate their symptoms? (e.g.; young children and older adults with cognitive decline).

22. Do concussions cause attention deficit hyperactivity disorder (ADHD)?

23. How effective is brain imaging (e.g., MRI) in confirming concussion, evaluating concussion severity, and predicting recovery from concussion?

24. How does sex and gender affect concussion susceptibility, severity of symptoms, and time to recover? Should treatment and prevention strategies be tailored to sex and gender to be most effective?

25. What is the most effective way to accurately identify and safely manage a suspected sports concussion at the time of injury?

26. How often does concussion result in cognitive impairment (i.e., loss of attention, loss of memory, feeling foggy), and what is the best way to treat this?

27. What protective equipment (e.g., helmets, face masks, head gear, mouth guards) is most effective in preventing concussion in sports?

28. How often do concussions result in dementia and/or Chronic Traumatic Encephalopathy (CTE)? (CTE is a progressive brain condition that occurs after repeated blows to the head). How can these long-term effects be best prevented or treated?

29. How effective is acupuncture in treating concussion symptoms?

30. What is the effectiveness of meditation and other mindfulness practices on concussion recovery?

31. Are there medications that can improve concussion recovery?

32. What is the best way to diagnose concussion in the emergency department?

33. How can the training of front-line doctors and other healthcare providers to recognize, diagnose and manage concussion be improved and kept up to date?

34. What is the most effective way to reduce the number of concussions occurring in a workplace?

35. What is the most effective tool (e.g., website, app, paper-based instructions) to help people with concussion adjust their day-to-day activities to manage symptoms?

36. After a concussion, what is the best approach for a return to physical activity, exercise, and sports (i.e., timing, type, and intensity of activity) to give the best outcome?

37. At what age should full body contact be introduced in contact sports and what strategies should be implemented to prevent concussions caused by full body contact?

© 2023 Osmond MH et al. *JAMA Network Open.*

38. What genetic factors influence the risk of concussion, prolonged symptoms, and long-term effects of concussion?

39. What are the long-term effects of single or multiple concussions, and how do these effects impact day-to-day life?

40. How does sharing information with a concussion patient about their prognosis affect the patient's symptoms and outcomes?

41. What are the cumulative, long-term effects of multiple minor impacts to the head, or very mild concussions, on the brain and how does this effect day-to-day life?

42. What is the best way to assess and treat dizziness and balance problems after concussion?

43. What types of physical and cognitive rest have the most positive impact on recovery from concussion, and what is the optimal duration of the rest? What specific activities worsen concussion symptoms and should be avoided?

44. What are the key strategies to ensure a safe, effective, and productive return to work for people recovering from a concussion?

45. What is the most effective way of differentiating prolonged concussion symptoms (known as post-concussion syndrome) from symptoms that are similar but unrelated to the concussion?

46. To what extent does the age at which a concussion occurs (i.e., child, adolescent, adult, older adult) impact the severity of symptoms and time to recover? Should treatment and prevention strategies be tailored to age to be most effective?

47. What is the role of physical examination tests (e.g., eye movement, balance and coordination tests) in accurately and objectively diagnosing a concussion and predicting its severity?

48. How effective are cannabis products (cannabinoids such as CBD) in treating specific symptoms of concussion?

49. What is the effectiveness of early referral and treatment by a concussion specialist team (with combined medical assessment, physical therapies, and mental health support) on concussion outcomes and length of recovery?

50. How common are hearing problems (i.e., sound hypersensitivity or tinnitus (ringing in the ears)) after concussion and how are these best treated?

51. Do concussion outcomes improve by increasing public awareness of: 1) the signs and symptoms of a concussion; 2) the seriousness of a concussion; and/or, 3) when and where to seek care following a concussion?

52. What is the best way to reduce the number of concussions suffered by students while at school? What is the most effective way to train school personnel to recognize symptoms of concussion and provide guidance to students with concussion?

53. How effective are physical manipulation therapies, performed by physiotherapists, osteopathic physicians and/or chiropractors, in the treatment of concussion?

54. After a concussion, what is the best timing and approach to return to learning activities and educational settings (e.g., school or university) to give the best outcome?

© 2023 Osmond MH et al. *JAMA Network Open.*

55. What is the correlation between concussion severity and disturbed sleep patterns? What tools, aids, medications, or other interventions are most effective in managing these sleep disturbances?

56. What is the best way to manage light sensitivity caused by a concussion?

57. What is the effect of socioeconomic and cultural factors (e.g. race, income, sexual orientation, language, housing, social/community connection) on the diagnosis, management and prognosis of concussion?

58. What factors or tests best predict a prolonged recovery from concussion (known as post-concussion syndrome), and how can this information be used to develop a tailored strategy to manage the symptoms and support recovery?

59. How important is proper nutrition in managing concussion symptoms and are there benefits to taking nutritional supplements (e.g., vitamins)?

© 2023 Osmond MH et al. *JAMA Network Open.*
