# Supplementary material for: Partnering With Patients, Caregivers, and Clinicians to Determine Research Priorities for Concussion
Source: JAMA Netw Open. 2023 Jun 7;6(6):e2316383. doi: 10.1001/jamanetworkopen.2023.16383 (PMC10248744; doi:10.1001/jamanetworkopen.2023.16383)
Supplement: Supplement 2. — Nonauthor Collaborators. Canadian Traumatic Brain Injury Research Consortium [file jamanetwopen-e2316383-s002.pdf]

Supplemental Online Content: Nonauthor Collaborators
*First name, last name, and suffix (if applicable) are required and will appear in PubMed.

*Group Name(s): Canadian Traumatic Brain Injury Research Consortium (CTRC)							
***First Name and Middle Initial(s)**	***Last Name**	***Suffix (eg, Jr, III)**	**Academic Degrees**	**Institution**	**Location (city, state/province, country)**	**Role or Contribution, eg, chair, principal investigator**	**Group (if more than 1 Group listed in the byline) and/or Subgroup (eg, Steering Committee)**
Mark	Bayley		MD	Toronto Rehabilitation Institute and University of Toronto	Toronto, ON, Canada	Co-Investigator	Canadian Traumatic Brain Injury Consortium (CTRC)
Phil	Fait		PhD	Université du Québec à Trois-Rivières	Trois-Rivières, QC, Canada	Co-Investigator	Canadian Traumatic Brain Injury Consortium (CTRC)
Isabelle	Gagnon		PhD	Montreal Children’s Hospital and McGill University Health Center	Montreal, QC, Canada	Co-Investigator	Canadian Traumatic Brain Injury Consortium (CTRC)
Noah D	Silverberg		PhD	University of British Columbia, and GF Strong Rehab Centre	Vancouver, BC, Canada	Co-Investigator	Canadian Traumatic Brain Injury Consortium (CTRC)
