# Supplementary material for: Partnering With Patients, Caregivers, and Clinicians to Determine Research Priorities for Concussion
Source: JAMA Netw Open. 2023 Jun 7;6(6):e2316383. doi: 10.1001/jamanetworkopen.2023.16383 (PMC10248744; doi:10.1001/jamanetworkopen.2023.16383)
Supplement: Supplement 3. — Data Sharing Statement [file jamanetwopen-e2316383-s003.pdf]

# Data Sharing Statement

Osmond. Partnering With Patients, Caregivers, and Clinicians to Determine Research Priorities for Concussion. *JAMA Netw Open*. Published June 07, 2023. doi:10.1001/jamanetworkopen.2023.16383

**Data**
**Data available:** Yes
**Data types:** Deidentified participant data
**How to access data:** osmond@cheo.on.ca, https://www.jla.nihr.ac.uk/priority-setting-partnerships/concussion-canada/ (to be uploaded after publication)
**When available:** With publication

**Supporting Documents**
**Document types:** None

**Additional Information**
**Who can access the data:** The data will be available to anyone requesting the data
**Types of analyses:** For any purpose
**Mechanisms of data availability:** Without investigator support
